# Supplementary material for: Developing a Machine-Learning ‘Smart’ PCR Thermocycler, Part 1: Construction of a Theoretical Framework
Source: Genes (Basel). 2024 Sep 11;15(9):1196. doi: 10.3390/genes15091196 (PMC11431463; doi:10.3390/genes15091196)
Supplement: Supplementary file 1 [file genes-15-01196-s001.zip › genes-3180224-supplementary.pdf]

SUPPLEMENTARY MATERIAL:

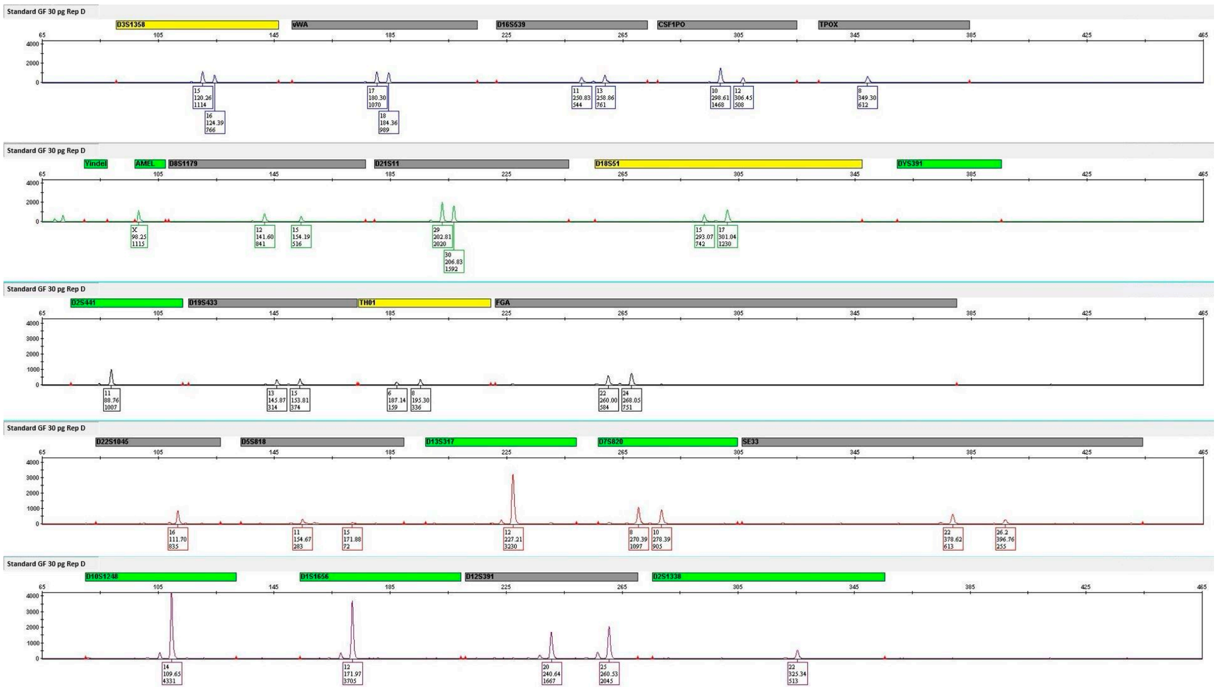

Supplementary Figure 1: STR profile produced using 30 pg of control DNA following the standard GlobalFiler^{TM} manufacturer's protocol. Replicate D electropherogram taken from GeneMapper-IDX (version 1.4).

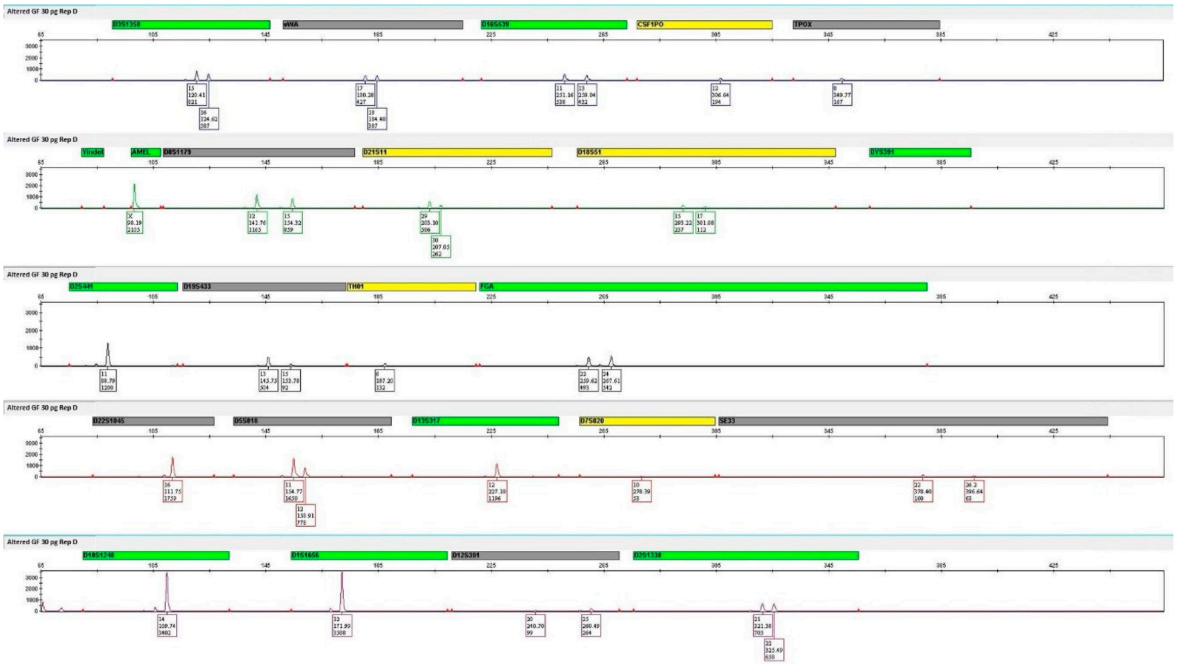

Supplementary Figure 2: STR profile produced using 30 pg of control DNA using the modified GlobalFiler^{TM} protocol. Replicate D electropherogram taken from GeneMapper-IDX (version 1.4).

Supplemetary Table 1: The number of observed alleles, percent allele loss and sub-source likelihood ratios for all STR profiles produced from 30 pg of starting material using the combined GlobalFiler and Investigator Quantiplex Pro reagents for each PCR program trialled. For all profiles the expected number of donor alleles was 37. Replicates that did not meet the requirements for upload to the National Criminal Identification Database (NCIDD) have been marked with an asterisk (*).

PCR Program	Replicate	Number of Observed Alleles	Average Percent Allele Loss (%)	Sub-Source Likelihood Ratio
Standard GlobalFiler™ manufacturer's protocol	A	32	13.51	9.09×10^{19}
	B	31	16.22	9.04×10^{17}
	C	32	13.51	1.71×10^{18}
	D	36	2.70	3.88×10^{23}
	E	36	2.70	4.04×10^{24}
Primed standard GlobalFiler™ manufacturer's protocol	A	24	35.14	1.81×10^{14}
	B	27	27.03	1.63×10^{10}
	C	6*	83.78	7.77×10^{2}
	D	26	29.73	1.80×10^{18}
	E	21	43.24	2.35×10^{15}
Modified GlobalFiler™ protocol	A	34	8.11	2.97×10^{15}
	B	29	21.62	1.54×10^{18}
	C	31	16.22	1.67×10^{17}
	D	34	8.11	1.82×10^{21}
	E	32	13.51	5.00×10^{18}
Primed modified GlobalFiler™ protocol	A	16	56.76	2.71×10^{7}
	B	9*	75.68	4.63×10^{5}
	C	16	56.76	2.87×10^{7}
	D	20	45.95	3.11×10^{9}
	E	3*	91.89	6.00×10^{-4}

Supplemetary Table 2: The breakdown of profile quality scores assigned to all STR profiles produced from 30 pg of starting material using the combined GlobalFiler and Investigator Quantiplex Pro reagents for the various PCR programs trialled.

**PCR Program**	**Rep**	**Peak Heights (RFU)**			**Penalties**			**Profile Quality Score**
		Mean	***Std. Dev.***	***COV***	***Peak Height***	***COV***	**Artefacts**	
Standard GlobalFiler™ manufacturer's protocol	A	675	663	0.98	-7.46	-2.13	0	-13.00
	B	706	820	1.16	-7.40	-2.52	0	-13.32
	C	570	494	0.87	-7.66	-1.88	0	-12.99
	D	812	643	0.79	-7.20	-1.72	0	-12.33
	E	675	535	0.80	-7.47	-1.73	0	-12.60
Primed standard GlobalFiler™ manufacturer's protocol	A	155	292	1.88	-8.50	-4.10	-8	-22.64
	B	242	360	1.49	-8.31	-3.23	-4	-18.37
	C	15	30	2.04	-8.80	-4.42	-8	-24.23
	D	181	360	2.23	-8.44	-4.95	-6	-20.91
	E	133	348	2.63	-8.54	-5.70	0	-14.60
Modified GlobalFiler™ protocol	A	334	321	0.96	-8.13	-2.08	0	-13.61
	B	320	330	1.03	-8.15	-2.24	0	-13.80
	C	302	296	0.98	-8.19	-2.13	0	-13.72
	D	548	521	0.95	-7.70	-2.06	0	-13.17
	E	677	638	0.94	-7.46	-2.05	0	-12.90
Primed modified GlobalFiler™ protocol	A	84	255	3.05	-8.65	-6.62	-10	-23.46
	B	33	66	1.98	-8.76	-4.29	-10	-26.75
	C	74	120	1.62	-8.67	-3.53	-6	-21.76
	D	108	138	1.28	-8.60	-2.77	-6	-20.84
	E	5	15	3.26	-8.82	-7.07	-6	-25.07
